# Identification of retention strategies for neurosurgeons in Iran: Results from expert panels

**Published:** 2017-04-04

**Authors:** Sima Rafiei, Sina Abdollahzadeh, Fariba Hashemi, Mohammad Ranjbar

**Affiliations:** 1 Department of Health Management, School of Health, Qazvin University of Medical Sciences, Qazvin, Iran; 2 Department of Surgery, School of Medicine, Qazvin University of Medical Sciences, Qazvin, Iran; 3 Department of Operating Room, School of Paramedicine, Qazvin University of Medical Sciences, Qazvin, Iran; 4 Health Policy Research Center, School of Public Health, Shahid Sadoughi University of Medical Sciences, Yazd, Iran

**Keywords:** Retention, Strategy, Health Human Resource, Health Professional, Health Manpower, Neurosurgeon, Workplace

## Abstract

**Background:** The key challenge is how to encourage and retain health professionals in their work location. There is a list of policy options for this purpose but applying an appropriate and effective set of strategies requires a country level research. Our study aimed to identify retention strategies for neurosurgeons and examine both the importance and feasibility of the identified strategies using expert panels’ point of view.

**Methods:** First of all, a literature review was conducted to identify retention strategies for physicians. Then to gain consensus on the strategies and determine their importance and feasibility an expert panel was organized and a modified Delphi process was used.

**Results:** A total of 40 strategies were identified by the panel classified in seven categories of income and economic factors, professional/job factors, clinical infrastructure, personal/family factors, living condition and welfare, educational factors and career development, governmental regulations and management policies.

**Conclusion:** Based on the study results, three areas of economic incentives, personal and professional factors got the greatest priority in health professional planning for retention purposes.

## Introduction

Shortage of health professionals whether due to poor distribution or insufficient admission quota in the profession has become a significant problem in many countries. To resolve such problems, planning for health professionals has got a great importance.^1^ The aim is to ensure that health professionals are available and ready to provide services in “the right place at the right time with the right skills”.^2^^,^^3^

In almost all countries health professional-mainly physiciansare likely to work in developed urban cities with proper economic condition, better living facilities and advanced clinical infrastructure.^4^ This inclination would probably cause imbalanced concentration of health manpower in urban areas as well as continuing shortages in rural and remote communities.^5^

Such inequitable distribution in health human resources not only decreases the accessibility to care and negatively affects quality of healthcare services, but also causes extra costs due to additional recruitment and replacements.^6^ Malfunction in maintaining health professionals in places where they are most needed has been mentioned as the key reason for the occurrence.^7^

Almost all governments endeavored to provide an evidence based solution to attract physicians and improve their retention in different areas of the country.^8^ Some focused on monetary incentives and some on nonfinancial motivations.^9^ In this regard, World Health Organization has introduced 16 retention strategies such as regulatory policies, financial incentives, educational opportunities, and personal and professional inducement plans.^10^^,^^11^

Literature confirmed that successfulness of strategies was highly reliant on the country setting and type of health workers.^12^ In Iran, about 2200 specialists are working in 270 underserved areas who generally are not satisfied with their income and work environment. Most of them do not ask for continuing to serve in remote areas and try to find an opportunity to leave as soon as possible.^13^ To provide an appropriate feedback toward similar dilemmas, effective strategies for improving physicians’ retention in such areas could be significantly helpful.^14^^,^^15^

On May 2014, a national program “Evolution in Health System of Iran” was established and obligated to be performed headed for overcoming obstacles to access health care services and increase the opportunity to obtain high quality and timely services among people. One of the main objectives of the program was to guarantee an equitable distribution of health professionals within a country and improve their retention in work places. 

Moreover, need for neurosurgery services has dramatically increased due to an elderly population, inactive life styles among Iranian people and principal rise in road accident rates. Neurosurgeons play important role in diagnosis, treatment and rehabilitation of patients suffering from neurospinal disorders or injuries.^16^^,^^17^ Thus planning for appropriate strategies to increase the probability of neurosurgeons’ retention in underserved areas of the country has got a significant importance to properly respond population health needs.^18^

Literature emphasized that salary increase, improvement in quality of health facilities and opportunity for continued education and career development were some of the intervention policies to persuade health workforce maintain in their workplace. Research conducted in Ethiopia and Ghana also mentioned provision of superior living condition and welfare as one of the most imperative elements for health professionals to accept job retention.^19^ Similarly, educational scholarships and career development opportunities were ranked as significant job profiles by most of the health workers.^20^ Hanson and Jack declared that improved housing, adequate medical equipment and reduced time commitment were other key factors in physicians’ decision to retain.^19^ Findings of study conducted by Rafiei, et al.^21^ revealed that neurosurgeons were ready to give up some amount of income in return to obtain subsidized housing and opportunity to have permission for dual practice. To work in large developed cities rather than rural areas, they also requested an increase in level of income. Authors added that mixtures of incentives could improve neurosurgeons' recruitment and retention in rural or remote areas.^21^ Therefore, knowing important and applicable policy interventions could be beneficial for policy makers to better decide based on it. 

To identify such applicable strategies, we conducted a study to determine retention strategies for neurosurgeons and examine both the importance and feasibility of the identified strategies using expert panels’ point of view.

## Materials and Methods

Study has been conducted in two phases: 1) literature review and 2) expert panel. 

***Identification of retention strategies: ***A literature review was done to identify retention strategies for neurosurgeons. Keywords used to find out relevant researches done in Medline, Ovid and Google Scholar (2000 to 2015) included: "health human resources or health professionals or health manpower", "neurosurgeons or neurosurgery specialists", "retention strategies", "workplace or place of activity”. From the literature review, 85 strategies relevant to retention issues in various medical specialties were identified.

Strategies were assessed by two members of the study group for replication, transparency, applicability and compatibility with country circumstances. Some of them were omitted, revised or reworded to comply with the conditions related to neurosurgery. This investigation resulted in 40 retention strategies categorized into seven themes: income and economic factors (n = 8), professional/job factors (n = 6), clinical infrastructure (n = 5), personal/family factors (n = 9), living condition and welfare (n = 5), educational factors and career development (n = 3), and governmental regulations and management policies (n = 4). 

***First stage of the expert panel: Rating the strategies: ***Members of the expert panel were selected among planners and policy makers in health human resource planning and establishers of retention policy package among physicians and specialists in form of health system reform. Panel composition based on individual’s expertise and affiliated organization is shown in table 1. Finally, 14 panelists were chosen to participate in the study which was large enough to allow for variety of perspectives.

A Modified Delphi technique was used to implement the expert panel process.^22^ In the first step, 40 strategies identified from the literature were sent to the panelists. Then they were asked to rank the strategies using a Likert type scaling system ranging from “minimum” (1) to “maximum” (9), based on two aspects of importance and feasibility. Importance was defined as how suitable and helpful the strategy could be for health human resource planning especially in the field of neurosurgery, while feasibility presented the rationality and applicability of the strategy. Results of the first round were consequently used as inputs for the second phase of the panel. Importance and feasibility rankings were entered in to a spreadsheet to conduct a descriptive analysis. Strategies that had been scored between 7-9 from both aspects of feasibility and importance by almost all panelists were considered high rating, those with a combination of 4-9 in any of the two facets were considered medium rating, while strategies rated between 1-3 were regarded as low rated ones.^23^

***Second stage of the expert panel: A face to face meeting: ***After providing a list of rated strategies, a meeting with the presence of all panelists was held to reach an agreement and make final decisions. In the meeting, participants discussed on medium rated strategies since it was already agreed on high and low rated ones. In fact, strategies belonged to this group were rated for the second time. At this point, each expert had an opportunity to work on transparency, better understanding and applicability of the strategies.

## Results

Results of scaling 40 retention strategies revealed that panelists had agreed on 25 strategies as high rating, 10 strategies as high/medium and 5 as medium rating. Among these, the most important and applicable strategy group was mentioned to be income and economic factors. Afterward job/professional, personal/family and educational factors got the greatest importance from the participants’ point of view (Table 2).

In the second phase of the panel, participants were asked to review high/medium- and medium-rated strategies to re-rank them. Table 3 shows a modification in experts’ opinion toward the rating of strategies in round II of the Delphi method. As data confirm, there was a 10% decrease in high/medium-rated strategies and 5% increase in those with medium and low rating.

Identification of each strategy’s rating from both perspectives of importance and applicability provides valuable information for policy makers to plan for physicians’ retention more effectively. Table 4 depicts a detailed description of average scores and total ranking of strategies.

As it is shown in table 2, timely fee for service payment to specialist has got the greatest priority while provision of an appropriate cooling or heating system had the least importance from the panelists’ viewpoint.

**Table 1:** Panel composition based on expertise and affiliated organization

**Expertise**	**Organization**	**Number of ** **participants**
Healthcare management	Curative Deputy of Ministry of Health Institute of health Sciences, Tehran University of Medical Sciences (TUMS)	6
Social medicine	Shahid Beheshti University of Medical Sciences	4
General practitioner	Curative Deputy of Ministry of Health	2
Neurosurgeons	Shariati Hospital, TUMS Trauma Research Center in Sina Hospital, TUMS	2

**Table 2:** Rated strategies in phase I

**Strategy group**	**Number of ** **strategies in each ** **group**	**High-rated ** **strategies ** **[n (%)]**	**High/medium-** **rated strategies ** **[n (%)]**	**Medium-rated ** **strategies ** **[n (%)]**
Economic factors	8	6 (75.0)	1 (12.5)	1 (12.5)
Professional factors	6	4 (66.6)	2 (33.4)	0 (0)
Clinical infrastructure	5	3 (60.0)	1 (20.0)	1 (20)
Personal factors	9	6 (66.6)	2 (22.3)	1 (11.1)
Living condition	5	2 (40.0)	2 (40.0)	1 (20)
Educational factors	3	2 (66.6)	1 (33.4)	0 (0)
Governmental regulations	4	2 (50.0)	1 (25.0)	1 (25.0)
Total	40	25 (62.5)	10 (25.0)	5 (12.5)

## Discussion

Study aimed to identify retention strategies to give an opinion to health human resource planners for specialists’ retention working in Iran. Similar to the literature findings, our study revealed that combination of different monetary and nonmonetary incentives could improve neurosurgeons' retention in their workplace.^24^ Results also emphasized on four main important issues in developing a competitive plan for physicians’ retention including financial incentives, professional, personal and educational factors.

***Economic factors/financial incentives: ***In many countries, the most influential factor for retaining health manpower in their workplace is financial incentives.^25^ To achieve higher income levels, health workers especially physicians try to look for an opportunity to supplement their income through dual practice.^26^ In fact to consider an attractive job profile, physicians are willing to have opportunity for dual practice and consequently gain higher levels of salary. Literature found out that the impact of income and monetary factors on physicians’ retention ranged between 20% in USA^28^ to 86% in Australia.^27^

***Job/professional factors: ***Similar to our findings, literature confirmed that health workers looked beyond financial incentives and considered improvement in professional factors and quality of work environment as important retention strategies. Supportive management, team work, shared decision making about practice management, and open communication with supervisors are among strategies to improve working condition.^29^ In a qualitative study among 16 nurses working in health centers of western Canada, study participants expressed their desire for improved teamwork and effective communication with members of clinical team.^30^ Also important was adequate, trained and skilled auxiliary workforce such as nurses, physiotherapists and Anesthesiologists to give the feeling of working in a supportive environment to physicians.^31^

Numerous studies have regarded poor working atmosphere, lack of supportive management, poor job direction and inappropriate workload as key deterrents to attract or retain in a workplace.^32^^-^^34^

Maintaining safety was another strategy that could reduce violence and hostility in a work environment. Physicians support plan and provision of adequate number of guards in the workplace have been mentioned as related strategies to improve safety. Some studies have also emphasized on trigger role of administrative or managerial positions offered to physicians.^35^

**Table 3:** Rated strategies in phase II

**Strategy group**	**Number of ** **strategies in ** **each group**	**High-rated ** **strategies ** **[n (%)]**	**High/medium-** **rated strategies ** **[n (%)]**	**Medium-rated ** **strategies ** **[n (%)]**	**Low-rated ** **strategies ** **[n (%)]**
Economic factors	8	6 (75.0)	1 (12.5)	1 (12.5)	0 (0)
Professional factors	6	4 (66.8)	1 (16.6)	1 (16.6)	0 (0)
Clinical infrastructure	5	3 (60.0)	1 (20.0)	1 (20.0)	0 (0)
Personal factors	9	6 (66.7)	1 (11.1)	1 (11.1)	1 (11.1)
Living condition	5	2 (40.0)	1 (20.0)	1 (20.0)	1 (20.0)
Educational factors	3	2 (66.7)	0 (0)	1 (33.3)	0 (0)
Governmental regulations	4	2 (50.0)	1 (25.0)	1 (25.0)	0 (0)
Total	40	25 (62.5)	6 (15.0)	7 (17.5)	2 (5.0)

**Table 4:** Total ranking of retention strategies

**Strategies**	**Overall priority**		
	**Applicability**	**Importance**	**Total**
Timely fee for service payment	7.8	8.5	1
Distribution of physicians based on home prefectures	8.2	8.0	2
Supportive management	8.0	8.1	3
Permission for dual practice	7.8	8.2	4
Promotion of physical and clinical infrastructure	7.8	8.0	5
Promotion of educational facilities	7.8	8.0	6
Increase in full time payment	7.5	8.0	7
Employment opportunity for spouses	7.5	8.0	8
Opportunity for educational promotion	7.5	7.8	9
Reinforcement of family physician plan	7.0	8.0	10
Educational facilities for children	7.0	7.8	11
Improvement of neurosurgery equipment in hospitals	7.2	7.5	12
Additional quota for specialty acceptance	7.0	7.5	13
Competitive agreements with companies of high quality medical equipment	7.0	7.5	14
Clear regulation for physicians’ distribution system	7.1	7.3	15
Long-term and low interest loan to open a clinic	7.0	7.4	16
Long-term and low interest loan to establish an image center	7.0	7.3	17
Subsidized housing	7.0	7.2	18
Improvement of para clinical infrastructure	7.0	7.2	19
Offer managerial position	7.0	7.2	20
Suitable transportation system	7.0	7.1	21
Increase fee for service payments	7.0	7.1	22
Transparent job contracts	7.0	7.0	23
Support team work	7.0	7.0	24
Respect for religion, culture and ethnicity	7.0	7.0	25
Supportive regulation	6.0	7.0	26
Improvement of rehabilitation system	6.0	7.0	27
Appreciation system	6.0	7.0	28
Proper workload	5.1	7.5	29
Safety provision	6.0	6.1	30
Recreational facilities	5.0	7.1	31
Allocation of monthly fixed payment	5.0	7.0	32
Effective communication with ministry of health	6.0	5.6	33
Organizing tele-education system	6.0	5.5	34
Provision of essential drugs	5	5.4	35
Practice within same proximity to the site where residency training took place	5.4	4.6	36
Human resource reinforcement	4.2	4.4	37
Long term and low interest loan to buy a house	4.0	4.1	38
Physician distribution based on educational place	3.0	3.0	39
Provision of proper cooling or heating system	3.0	2.9	40

***Personal/family factors: ***Naturally, physicians like other humans have some personal needs which proper response to them could have a positive impact on work satisfaction. They expect to receive respect and recognition from the community. This sense of gratitude would satisfy their personal needs and encourage them to retain in a workplace.^36^^,^^37^ Addressing emotional needs such as proximity to family and providing working condition in an area that is compatible with their religious beliefs had also positive impact on their retention.^38^

***Educational factors: ***Study findings acknowledged that opportunity for continuous learning and career development was the fourth significant factor in physicians’ retention. Mangham and Hanson confirmed that nurses would sacrifice some pay increases to obtain the opportunity for continuous education.^39^ Continued education was also underpinned as key enthusiasm making rural jobs more attractive by Rockers, et al.^40^ A similar study conducted by Vujicic, et al. announced that possibility for long-term education and opportunity to exchange knowledge and experience with other colleagues could play an important role in increasing take up rate for rural jobs.^41^ Evidence also indicated that further training opportunities acted as important motivation to attract and retain health manpower in Malawi, Zambia, Uganda, Namibia and South Africa.^34^

## Conclusion

This is the first study conducted in Iran that provides a comprehensive list of retention strategies relevant to neurosurgeons arranged on the basis of importance and applicability. Study identified 40 strategies applicable for health human resource planners to retain physicians (neurosurgery specialists) in their workplace. Although the strategies with highest importance mainly focused on areas of income and economic enthusiasms, professional and work environment factors and personal incentives, other factors including living condition, clinical infrastructure, and governmental regulations also need to be considered in a planning process. Effective policy making in the field of health human resource retention requires inclusive and ongoing reviews of incentive methods to analyze their impact and select the most influential ones.

## Notes:

**How to cite this article:** Rafiei S, Abdollahzadeh S, Hashemi F, Ranjbar M. Identification of retention strategies for neurosurgeons in Iran: Results from expert panels. Iran J Neurol 2017; 16(2): 55-61.
